# Supplementary material for: Awareness regarding breast cancer amongst women in Pakistan: A systematic review and meta-analysis
Source: PLoS One. 2024 Mar 7;19(3):e0298275. doi: 10.1371/journal.pone.0298275 (PMC10919669; doi:10.1371/journal.pone.0298275)
Supplement: S2 File — This file contains the individual search strings used for each database to perform the literature search for this study. (PDF) [file pone.0298275.s002.pdf]

Supplementary File 2. Search strings used for database searches.

PubMed

(Pakistan)

AND

(breast cancer)

Filters
Time period: 2010-2023
Language: English

Embase

(‘Pakistan’)

AND

(‘breast cancer’/exp)

Filters
Time period: 2010-2023
Language: English

Scopus

“Pakistan**”**

AND

“breast cancer**”**

Filters
Time period: 2010-2023
Language: English

Google Scholar

(breast cancer)

AND

(Pakistan)

Filters
Time period: 2010-2023
Language: English
