# Supplementary material for: Awareness regarding breast cancer amongst women in Pakistan: A systematic review and meta-analysis
Source: PLoS One. 2024 Mar 7;19(3):e0298275. doi: 10.1371/journal.pone.0298275 (PMC10919669; doi:10.1371/journal.pone.0298275)
Supplement: S3 File — This file contains a detailed quality analysis using the National Institute of Health’s Quality Assessment Tool for Observational Cohort and Cross-Sectional Studies. (PDF) [file pone.0298275.s003.pdf]

Supplementary File 3. Quality analysis using the National Institute of Health's Quality Assessment Tool for Observational Cohort and Cross-Sectional Studies.

**Study (Overall Rating)**	1. Was the research question or objective in this paper clearly stated?	2. Was the study population clearly specified and defined?	3. Was the participation rate of eligible persons at least 50%?	4. Were all the subjects selected or recruited from the same or similar populations (including the same time period)? Were inclusion and exclusion criteria for being in the study prespecified and applied uniformly to all participants?	5. Was a sample size justification, power description, or variance and effect estimates provided?	6. For the analyses in this paper, were the exposure(s) of interest measured prior to the outcome(s) being measured?	7. Was the time-frame sufficient so that one could reasonably expect to see an association between exposure and outcome if it existed?	8. For exposures that can vary in amount or level, did the study examine different levels of the exposure as related to the outcome (e.g., categories of exposure, or exposure measured as continuous variable)?	9. Were the exposure measures (independent variables) clearly defined, valid, reliable, and implemented consistently across all study participants?	10. Was the exposure(s) assessed more than once over time?	11. Were the outcome measures (dependent variables) clearly defined, valid, reliable, and implemented consistently across all study participants?	12. Were the outcome assessors blinded to the exposure status of participants?	13. Was loss to follow-up after baseline 20% or less?	14. Were key potential confounding variables measured and adjusted statistically for their impact on the relationship between exposure(s) and outcome(s)?	**Overall**
Shoukat Z *et al.* 2023	Y	Y	Y	Y	N	NA	NA	NA	Y	NA	Y	NA	NA	Y	Good
Irfan R *et al.* 2021	Y	Y	Y	Y	Y	NA	NA	NA	Y	NA	Y	NA	NA	NA	Fair
Ullah Z *et al.* 2021	Y	Y	Y	Y	Y	NA	NA	NA	Y	NA	Y	NA	NA	Y	Good
Ali A *et al* 2020.	Y	Y	NR	Y	Y	NA	NA	NA	Y	NA	Y	NA	NA	N	Good
Naqvi AA *et al.* 2018	Y	Y	Y	Y	Y	NA	NA	NA	Y	NA	Y	NA	NA	Y	Good
Arif S *et al.* 2018	Y	Y	Y	Y	Y	NA	NA	NA	Y	NA	Y	NA	NA	CD	Fair
Rafique S *et al.* 2018	Y	Y	Y	Y	Y	NA	NA	NA	Y	NA	Y	NA	NA	NR	Fair
Sultana R *et al.* 2018	Y	Y	Y	Y	N	NA	NA	NA	Y	NA	Y	NA	NA	NA	Fair

Amin S *et al.* 2017	Y	Y	NR	Y	N	NA	NA	NA	Y	NA	Y	NA	NA	N	Fair
Masood I *et al.* 2016	Y	Y	Y	Y	Y	NA	NA	NA	Y	NA	Y	NA	NA	Y	Good
Noreen M *et al.* 2015	Y	Y	Y	Y	Y	NA	NA	NA	Y	NA	Y	NA	NA	N	Fair
Sarwar MZ *et al.* 2015	Y	Y	Y	Y	N	NA	NA	NA	Y	NA	Y	NA	NA	N	Good
Peltzer K *et al.* 2014	Y	Y	NR	Y	N	NA	NA	NA	Y	NA	Y	NA	NA	NA	Fair
Raza S *et al.* 2012	Y	Y	Y	Y	Y	NA	NA	NA	Y	NA	Y	NA	NA	NA	Good
Sobani ZU *et al.* 2012	Y	Y	Y	Y	Y	NA	NA	NA	Y	NA	Y	NA	NA	Y	Good
Khokher S *et al.* 2011	Y	Y	Y	Y	N	NA	NA	NA	Y	NA	Y	NA	NA	NA	Good
Ahmad S *et al.* 2011	Y	Y	Y	Y	Y	NA	NA	NA	Y	NA	Y	NA	NA	NA	Fair
Gilani SI *et al.* 2010	Y	Y	NR	Y	N	NA	NA	NA	Y	NA	Y	NA	NA	N	Good

Legend

Y	Yes
N	No
NA	Not Applicable
NR	Not Reported
CD	Could Not Determine
