# Supplementary material for: Awareness regarding breast cancer amongst women in Pakistan: A systematic review and meta-analysis
Source: PLoS One. 2024 Mar 7;19(3):e0298275. doi: 10.1371/journal.pone.0298275 (PMC10919669; doi:10.1371/journal.pone.0298275)
Supplement: S4 File — This file contains further meta-analysis results, including funnel plots, and tests for assessment of publication bias. (PDF) [file pone.0298275.s004.pdf]

Supplementary File 4. Further meta-analysis results.

Overall BCa Risk Factor Knowledge

Model: Binary Random-Effects Model
Metric: Proportion

Model Results

Estimate	Lower bound	Upper bound	Std. error	*P*-value
0.427	0.341	0.514	0.044	<0.001

Heterogeneity

tau^2	Q (df=14)	Het. p-Value	I^2
0.029	7145.600	<0.001	99.804

Funnel Plot

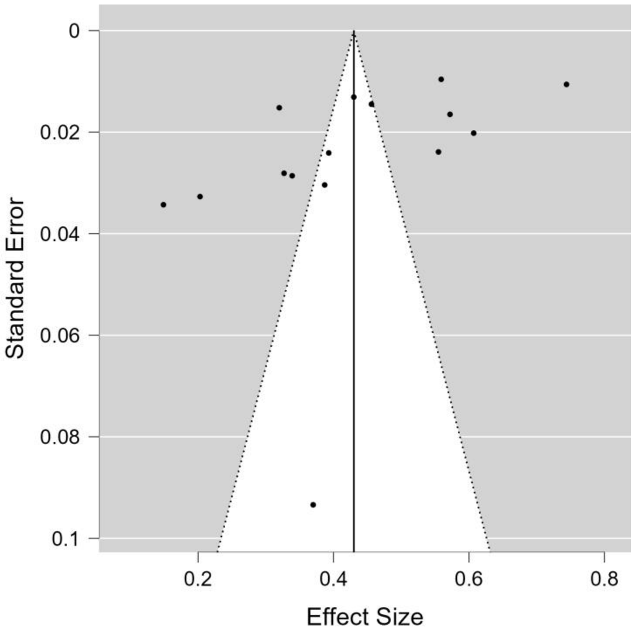

Funnel Plot Asymmetry

Rank Correlation Test for Funnel Plot Asymmetry		
	**Kendall's τ**	***p***
Rank test	-0.314	0.114
**Regression test for Funnel plot asymmetry ("Egger's test")**		
	**z**	***p***
**sei**	-1.783	0.075

Overall BCa Symptom Knowledge

Model: Binary Random-Effects Model

Metric: Proportion

Model Results

Estimate	Lower bound	Upper bound	Std. error	*P*-value
0.418	0.262	0.575	0.080	<0.001

Heterogeneity

tau^2	Q (df=7)	Het. p-Value	I^2
0.057	6021.729	<0.001	99.867

Funnel Plot

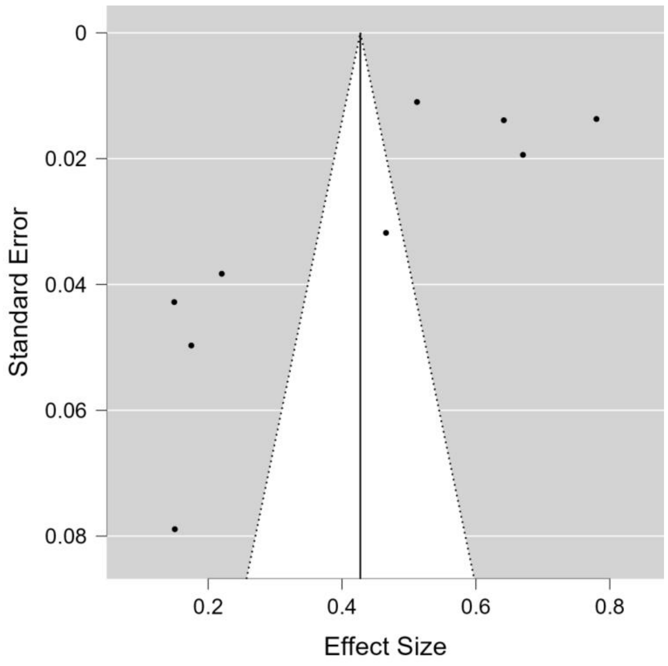

Funnel Plot Asymmetry

Rank Correlation Test for Funnel Plot Asymmetry		
	**Kendall's τ**	***p***
Rank test	-0.333	0.260
**Regression test for Funnel plot asymmetry ("Egger's test")**		
	**z**	***p***
**sei**	-3.954	<0.001

Overall BCa Diagnostic Modality Knowledge

Model: Binary Random-Effects Model
Metric: Proportion

Model Results

Estimate	Lower bound	Upper bound	Std. error	*P*-value
0.363	0.231	0.494	0.067	<0.001

Heterogeneity

tau^2	Q (df=10)	Het. p-Value	I^2
0.049	2908.041	<0.001	99.656

Funnel Plot

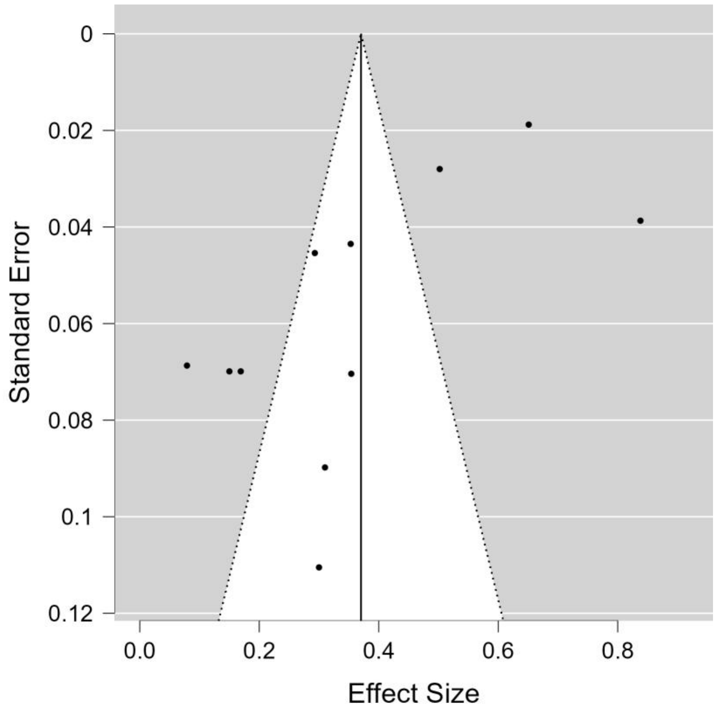

Funnel Plot Asymmetry

**Rank Correlation Test for Funnel Plot Asymmetry**		
	**Kendall's τ**	***p***
Rank test	-0.183	0.435
**Regression test for Funnel plot asymmetry ("Egger's test")**		
	**z**	***p***
**sei**	-2.511	0.012

Overall BCa Treatment Knowledge

Model: Binary Random-Effects Model
Metric: Proportion

Model Results

Estimate	Lower bound	Upper bound	Std. error	*P*-value
0.466	0.135	0.798	0.169	0.006

Heterogeneity

tau^2	Q (df=3)	Het. p-Value	I^2
0.114	2814.862	<0.001	99.893

Funnel Plot

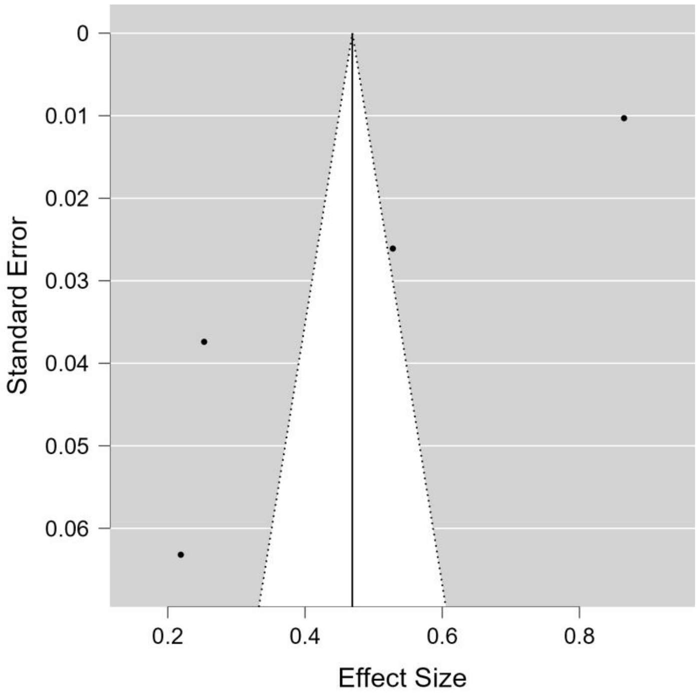

Funnel Plot Asymmetry

Rank Correlation Test for Funnel Plot Asymmetry		
	**Kendall's τ**	***p***
Rank test	-0.333	0.750
**Regression test for Funnel plot asymmetry ("Egger's test")**		
	**z**	***p***
**sei**	-3.509	<0.001

Overall Regular BSE Practice

Model: Binary Random-Effects Model
Metric: Proportion

Model Results

Estimate	Lower bound	Upper bound	Std. error	*P*-value
0.287	0.179	0.396	0.055	<0.001

Heterogeneity

tau^2	Q (df=10)	Het. p-Value	I^2
0.033	1130.933	<0.001	99.116

Funnel Plot

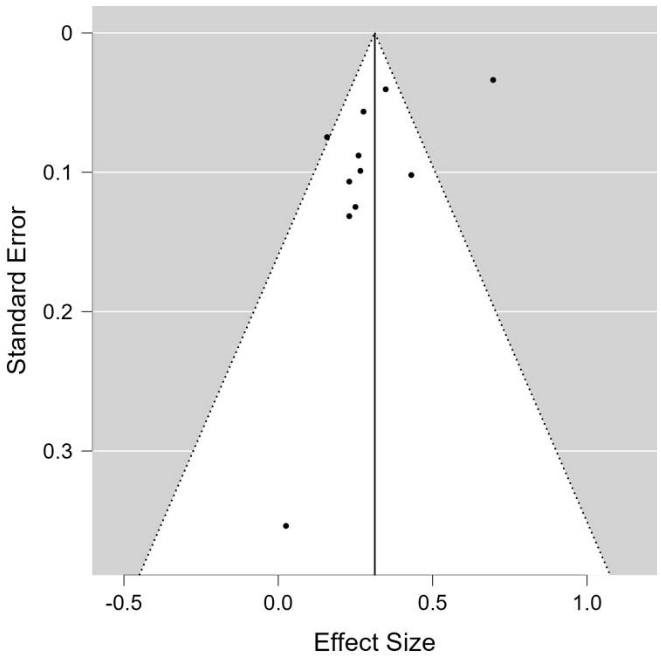

Funnel Plot Asymmetry

Rank Correlation Test for Funnel Plot Asymmetry		
	**Kendall's τ**	***p***
Rank test	0.309	0.218
**Regression test for Funnel plot asymmetry ("Egger's test")**		
	**z**	***p***
**sei**	-1.439	0.150

Ever Undergone CBE

Model: Binary Random-Effects Model
Metric: Proportion

Model Results

Estimate	Lower bound	Upper bound	Std. error	*P*-value
0.153	0.112	0.194	0.021	< 0.001

Heterogeneity

tau^2	Q (df=6)	Het. p-Value	I^2
0.003	119.415	< 0.001	94.976

Funnel Plot

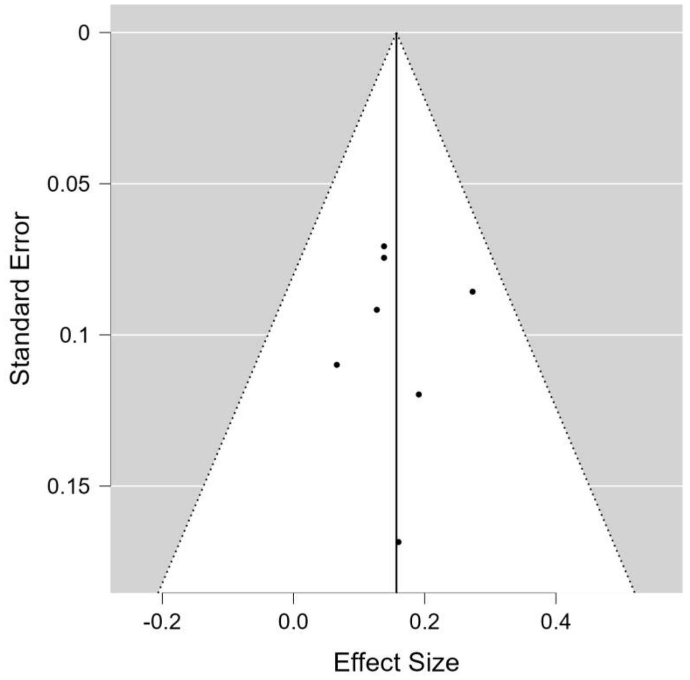

Funnel Plot Asymmetry

Rank Correlation Test for Funnel Plot Asymmetry		
	**Kendall's τ**	***p***
Rank test	0.048	1.000
**Regression test for Funnel plot asymmetry ("Egger's test")**		
	**z**	***p***
**sei**	-0.029	0.977
