# Supplementary material for: Awareness regarding breast cancer amongst women in Pakistan: A systematic review and meta-analysis
Source: PLoS One. 2024 Mar 7;19(3):e0298275. doi: 10.1371/journal.pone.0298275 (PMC10919669; doi:10.1371/journal.pone.0298275)
Supplement: Attachment — Submitted filename: Comments for authors.docx [file pone.0298275.s005.docx]

Dear Authors

Thank you for submitting the article for the possible consideration for publication. The area of your research was interesting and may contribute to the available knowledge of breast cancer which is one of the prevalent and high risks for most of women in the world. However, the following concerns are raised to improve the article.

Comments by sections

1. Title

Awareness Regarding Breast Cancer amongst Women in Pakistan: A Systematic Review and Meta-Analysis.

-Better to use status/level of awareness?

2. Abstract

-Not use abbreviation on abstract part

-Is knowledge and awareness similar? Your title was awareness but the associated factors mentioned for awareness. Make them consistent

-It lacks recommendation

Introduction

- I think you have three objectives. But, nothing mentioned on your introduction parts regarding your objectives. For example, regarding risk factors, treatment, attitude, screening practices.
- Please re-write the introduction parts magnitude, burden, risk factors, possible interventions and major gaps.

**Materials and methods**

Inclusion and exclusion criteria

- What about knowledge of breast cancer. Were they excluding form the search?
- Why you exclude the medical students/ physician. You are searching for awareness of BCa. Please elaborate the reason.
- On your document you used knowledge and awareness interchangeably. Please make it consistent.
- You said observational studies. Which means your study include case-control, cohort and cross-sectional. Please specify observational.

**Discussion**

- Please see again your discussion parts .it only focus on magnitude rather than factors.
