# Supplementary material for: Awareness regarding breast cancer amongst women in Pakistan: A systematic review and meta-analysis
Source: PLoS One. 2024 Mar 7;19(3):e0298275. doi: 10.1371/journal.pone.0298275 (PMC10919669; doi:10.1371/journal.pone.0298275)
Supplement: Attachment — Submitted filename: Comments for authors - 2.docx [file pone.0298275.s007.docx]

Dear Authors

Thank you for submitting the article for the possible consideration for publication. The area of your research was interesting and may contribute to the available knowledge of breast cancer which is one of the prevalent and high risks for most of women in the world. All comments were addressed. But, still your manuscript needs some grammar correction issue.

- You mentioned on response cross sectional study. But, throughout the manuscript I didn’t seen cross sectional study. So, please specify observational.
