# Supplementary material for: Awareness regarding breast cancer amongst women in Pakistan: A systematic review and meta-analysis
Source: PLoS One. 2024 Mar 7;19(3):e0298275. doi: 10.1371/journal.pone.0298275 (PMC10919669; doi:10.1371/journal.pone.0298275)
Supplement: Attachment — Submitted filename: Response to Reviewers (Round Two).docx [file pone.0298275.s008.docx]

**PONE-D-23-38477	Awareness Regarding Breast Cancer Amongst Women in Pakistan: A Systematic Review	and Meta-Analysis**

**Response to Reviewers’ Comments**

**Round Two**

15^th^ of January, 2024

Once again, I would like to begin by first thanking the reviewers who dedicated time out of their busy schedules towards our work.

Secondly, I would like to convey a description of the format of this document so as to make it easy, convenient, and smooth for the editor and the reviewers to understand how the comments have been addressed. Each reviewer’s comment is followed by a response. The answers highlight line numbers, and in quotations, the change that was made. I have also made use of evidence through links to support my responses where necessary.

**Responses to Comments by Reviewer #1**

*Comment 1*

Thank you for submitting the article for the possible consideration for publication. The area of your research was interesting and may contribute to the available knowledge of breast cancer which is one of the prevalent and high risks for most of women in the world. All comments were addressed. But, still your manuscript needs some grammar correction issue.

• You mentioned on response cross sectional study. But, throughout the manuscript I didn’t seen cross sectional study. So, please specify observational.

*Response 1*

I believe that perhaps some oversight has been made, as this comment was addressed in the first round of revisions. Nonetheless, I will point out the locations where we already specify the type of observational study design that was included In our research in order to provide maximum aid and convenience.

We specify the type of observational study design on the following line numbers:

1. Line 91, Methods>Inclusion/exclusion criteria: “We included observational ***cross-sectional studies*** that were published in/after 2010…”
2. Line 151. Results>Characteristics of Included Studies: “All included studies were observational ***cross-sectional studies***”
3. Line 163, Table 1, where each study is specified as having a cross-sectional study design.

**Responses to Comments by Reviewer #2**

No comments have been raised by Reviewer #2 in this round.

I hope my responses is satisfactory for Reviewer 1. Incase further explanations are needed or suggestions are available, I would gladly receive them. I want to once again thank both reviewers for their comments.

Warm Regards,

Dr. Abdul Rehman
